# The importance of deep phenotyping PH registries with a focus on the PVRI-GoDeep registry

**DOI:** 10.21542/gcsp.2020.12

**Published:** 2020-04-30

**Authors:** Paul A Corris

**Affiliations:** Professor of Thoracic Medicine, Translational and Clinical Science, Faculty of Medical Sciences, Newcastle University, UK

## Introduction

Although pulmonary hypertension has long been recognised to complicate many common diseases, especially left-sided heart disease and lung disease, most basic, translational and clinical scientists together with the pharmaceutical industry have, to date, focused predominantly on pulmonary arterial hypertension and chronic thromboembolic pulmonary hypertension when developing effective treatments^1^. Both entities are rare, leading to the erroneous belief that pulmonary hypertension in general is a rare condition, and not worthy of major global focus.

Whilst this focus on pulmonary arterial hypertension has led to the development of five classes of effective drug therapy, long term outcomes still remains poor for patients with pulmonary arterial hypertension and other classes of pulmonary hypertension. There is a clear need to identify new druggable targets and discover new or repurpose existing therapies if we are going to meet patient’s expectations. This is particularly important in patients with pulmonary hypertension associated with pre-existing cardiovascular or chronic respiratory disease, where the majority of existing drugs were found to be ineffective or even exert adverse effects.

The paradox is that some patients in these trials demonstrated improvements but not enough to satisfy statistical standards using group mean data. Moreover, these trials are expensive, of long duration and require many patients. The time has come to reconsider trial design, firstly basing patient selection on molecular phenotyping underpinning the mechanisms responsible for disease progression, irrespective of clinical phenotype. Secondly, clinical trial design should move on from the time-honoured, randomised controlled format using existing clinical outcomes to adaptive and enriched designs using biomarkers and novel end points.

Whilst to date, the true global burden of pulmonary hypertension remains unknown, it has certainly been underestimated. It can no longer be considered a rare disease and there is an urgent need to assess its impact on world health for a number of salient reasons.

Firstly, pulmonary hypertension is invariably associated with increased morbidity and mortality when occurring as idiopathic, hereditary or associated pulmonary arterial hypertension and pulmonary hypertension in association with cardiovascular and respiratory disorders. Secondly, the increasing age of populations worldwide is leading to a marked change in the distribution and phenotypes of patients presenting with pulmonary hypertension. A population-based study^2^ of 3381 participants in Rotterdam, Netherlands, reported echocardiographic signs suggestive of pulmonary hypertension in 2.6% of the overall population. The prevalence of echocardiographic signs of possible pulmonary hypertension was higher in older individuals.

Currently the reported incidence of idiopathic and hereditary pulmonary arterial hypertension, which has generally been thought to affect predominantly younger individuals and mostly females in the developed world is 1.1–7.6 per million adults per year and the prevalence of pulmonary arterial hypertension is 6.6–26.0 per million adults^3–5^.

Recent data from the USA and Europe^6–10^ demonstrates that pulmonary arterial hypertension is now frequently diagnosed in older patients, often with additional cardiovascular comorbidities. In the 2014 UK National Audit on Pulmonary Hypertension, the median age at the time of diagnosis of pulmonary arterial hypertension was 60 years and 29% of the patients were 70 years or older^11^. In Germany in 2014, the mean age of patients newly diagnosed with idiopathic pulmonary arterial hypertension was 65 years^8,10^.

Pulmonary hypertension associated with the left heart and respiratory disease will be mainly driven by a worldwide increase in life expectancy. In 2015, about 600 million people in the world were 65 years or older with a projected number of 700 million for 2020 and 1.6 billion for 2050^12^.

Accordingly, one can estimate that up to 50–70 million individuals, almost 1% of all people, are affected by pulmonary hypertension worldwide. This figure is expected to rise continuously over the next few decades as the global population enlarges and ages. With increasing life expectancy, individuals who reach the age of 40 might have a lifetime risk of one in ten of developing pulmonary hypertension. This risk is similar to the one in ten lifetime risk of developing COPD^13^ or the one in eight remaining lifetime risk for breast cancer in women of the same age^14^.

Pulmonary hypertension is thus not a rare disease and desperately requires novel therapies whose effectiveness is proven by novel clinical trials. Its global burden urgently needs to be determined and brought to the attention of the World Health Organisation, health care providers, and policy makers. Our patients, globally, deserve nothing less.

## Global registry

There is a pressing need to establish both a simple global registry and a deeply phenotyped registry of pulmonary hypertension, firstly to confirm the anticipated burden of disease and secondly to provide a basis for a new molecular classification of PH based on mechanisms of disease progression. This will be a vital means of identifying novel therapeutic targets and provide enriched patient populations that can take part in novel clinical trial designs.

PVRI has initiated a number of projects aiming to collect reliable data and establish a global map on the prevalence of PH and its variability among various regions around the world. To this end, close cooperation with global health organisations, such as the World Health Organization (WHO), the United Nations (UN), the NCD Alliance and the World Heart Federation (WHF), and organisations such as the Institute for Health Metrics from the University of Washington is part of PVRI politics. These activities aim to achieve recognition of PH as an important, high-prevalence global disease that is neglected largely at present.

Prior registry approaches are, however, impeded by the fact that the diagnosis of PH is uncertain when solely based on clinical criteria, that the best non-invasive screening technique (echocardiography) is not available in many regions and does itself have various substantial limitations, and that any assessment of PH classes/subgroups and deep patient phenotyping will not be possible. In the forthcoming period, sophisticated new imaging techniques, various *omic analyses performed in patient biomaterials and remote monitoring by novel wearable devices will dramatically increase our competence for deep phenotyping of PH patients. Therefore, PVRI has set up the PVRI Global Deep Phenotyping PH Meta-Registry (PVRI-GoDeep), with the following objectives:

1. Restrict data entrance to definite PH diagnosis based on right heart catheterisation.
2. Profit from established PH registries of PVRI members in all parts of the world, who remain proprietors of their registries but are willing to feed their data into this global “Meta-Registry”; support setup or further elaborate local registries of key regional centers if not available.
3. Demand a minimum common data trunk, adequate to allow reliable diagnosis of the various PH classes and subgroups, recording of further essential phenotypic data and assessment of treatment concepts and clinical follow-up.
4. Expand this trunk by facultative specialised modules collecting further deep phenotyping/genotyping data (genetics, epigenetics, *omics, advanced imaging, wearable device-based monitoring and/or miniaturised point-of-care self-testing), which may initially be fed only by subgroups of centers, but in the long run provide an invaluable basis to approach individualisation of PH treatment on a much more sophisticated basis than currently possible.
5. Establish a periodical electronic interface-based automated data update from the local registries into the Meta-Registry, thereby avoiding imposing any additional workload for the local registries once the interface is established.
6. Thereby provide PVRI with a) by far the largest deep phenotyping PH data bank worldwide (expect >25,000 patient data when fully established), and b) the only deep phenotyping PH data bank spanning over all continents, thereby offering insights into specific geographical and ethnical profiles of this disease. PVRI will thus develop into *the* go-to place for PH specialists and companies addressing pulmonary vascular diseases around the world.

To achieve these goals, adequate hard- and software tools will be employed for data harmonization and application programming interface (API) based automated data transfer and state of the art bioinformatic/statistical analysis.

**Figure 1.**
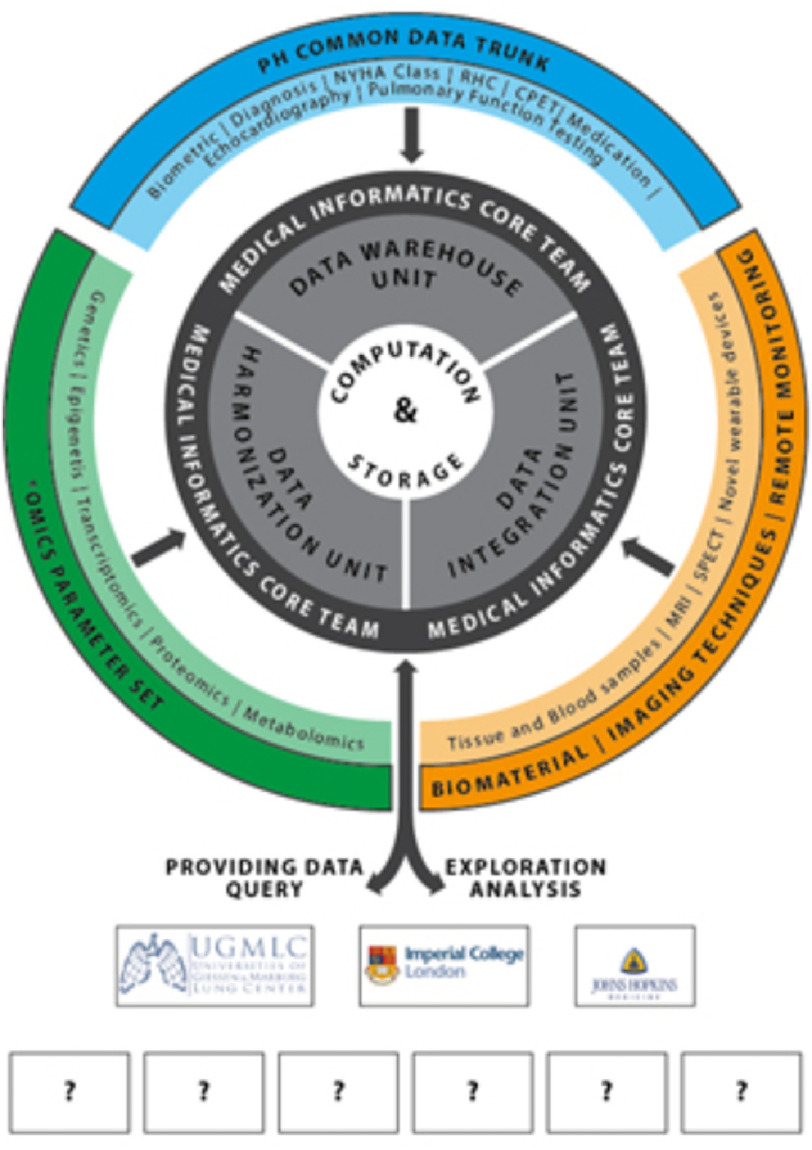
Organisation of the PVRI-GoDeep registry.

Each of the registries to be linked with PVRI-GoDeep will remain proprietor of its own data, but the scientists in charge will agree that the common data trunk (and further facultative modules) is fed into the central PH data repository provided by PVRI-GoDeep (Figure 1). This will be located at the University of Giessen in Germany.

PVRI-GoDeep operates under the responsibility of the University of Giessen Ethics Committee. A positive vote of this Ethics Committee is laid down before initiation of PVRI-GoDeep. Moreover, each local registry documents that the feeding of anonymised data into the central PVRI-GoDeep repository for unlimited storage (if not consent is withdrawn), central data analysis and data sharing within the PVRI-GoDeep Consortium as well as with PVRI-GoDeep clients from academia or industry, is approved by the respective local ethics committee and legal entity.

A state of the art bioinformatic/statistical tool set for analysis of the PVRI-GoDeep data set will be made available by the PVRI-GoDeep competence team. Moreover, the very large number of patients will enable the analysts to work very productively with novel systems biology and medicine approaches, predictive modeling, artificial intelligence and deep learning technologies. Combined with deep phenotyping, PVRI-GoDeep is expected to constitute a powerful toolbox to drive the development of individualised PH treatment and personalised medicine approaches beyond the currently available concepts.

Due to the large data set, cohorts for external validation are already included: external validation may be based on the different geographical areas or operate via splitting the original data set (total cohort) and using one part of the patients as development cohort and the other part as validation cohort (cross validation).

PVRI-GoDeep can easily be utilised for identification of possible study participants for clinical trials. Registry trials can be planned inside the registry. Normal values, prevalences and incidences of specific pathologies can be derived.

All types of scientific analysis of the PVRI-GoDeep data set will be decided by the PVRI-GoDeep Consortium which concurrently serves as Use and Access Committee (UAC) and works on the basis of predefined bylaws, respecting generally accepted data protection, ethics and compliance rules.

Each of the local registries feeding their data into PVRI-GoDeep will have a representative in the PVRI-GoDeep Consortium/UAC.

Each representative of the local registries may shape requests for scientific analysis of the global data pool, to be decided by the PVRI-GoDeep Consortium/UAC, free of charge.

All academia-based PVRI members may shape requests for scientific analysis of the global data pool, to be decided by the PVRI-GoDeep Consortium/UAC.

Academic organisations and pharmaceutical/commercial companies (third parties) will be charged a fee for access to data and/or analysis. Requests are to be decided by the PVRI-GoDeep Consortium/UAC.

## Added scientific value

Combining several current registries to an inter-continental Meta-Registry provides substantial added scientific value:

1. It provides the most comprehensive approach to assess the global burden of PH by extrapolating “high quality” data from reference centers spotted around the world.
2. Profiling of differences in PH categories among regions/countries/continents will be possible, referring to PH classification, demographics, disease severity, use of PH medication, etc.
3. Profiling of differences in PH categories among various ethnicities will be possible.
4. Childhood PH and early adult-onset PH may be compared with late adult-onset PH.
5. Assessment of regional differences in the “natural course” of the disease will be possible, as follow up data will be included in Go-Deep: findings may be linked to screening differences, regional levels of awareness of the disease, differences in the health care system and PAH specific drug availability, environmental factors, background therapies, use of/response to specific PH medication etc.
6. Leveraging the “large numbers” in GoDeep will allow asking questions beyond the scale of local registries, including “built-in” cohorts for cross-validation.
7. It will provide an invaluable international knowledge base for planning clinical trials.
8. It is expected to become *the* reference database for national and international (FDA, EMA) agencies as well as organizations with a focus on Global Health (and a recently stated interest in PVD) such as the WHO, NCDA, and the WHF, to name a few.

## Governance

PVRI-GoDeep is an entity of the PVRI. It is hosted at the Giessen University PH Center, Germany. Its bodies are the PVRI-GoDeep Consortium/UAC, a Coordinator’s Committee, the PVRI-GoDeep Competence Team and a Scientific Advisory Board. Details are laid down in the PVRI-GoDeep bylaws.

The PVRI-GoDeep Consortium/UAC is chaired by the Coordinator. Each local registry joining the PVRI-GoDeep Consortium and entering its data into the PVRI-GoDeep repository delegates one member and has one vote in the PVRI-GoDeep Consortium/UAC. This board decides on the rules of procedure for processing of data contained in the PVRI-GoDeep repository and on any request on PVRI-GoDeep data analysis by academic or commercial applicants. Decisions may be taken by way of written correspondence, during a face-to-face meeting, or by telephone/video conferencing. Decisions are made by majority vote.

## Conclusions

The development of a deeply phenotyped, multinational data base or registry of patients with PH represents a much-needed resource if we are going to make advances in understanding of the global impact of this condition, molecular drivers of the disease and the identification of much needed novel targets and therapeutic approaches.

The GoGlobal project of PVRI represents an ambitious attempt to fill the current void in this area and we look forward to its development and its potential for benefitting the whole PH community.
